# Decoding GNAO1 mutations using *Caenorhabditis elegans* model system: past approaches and future prospectives

**DOI:** 10.3389/fncel.2025.1633744

**Published:** 2025-07-23

**Authors:** Shubham Yadav, Satya Santoshi Veliventi, Somya Bhandari, Sakshi Gangurde, Shreeya Naik, Shraddha N. Bhagwat, Santosh Kumar

**Affiliations:** ^1^Biotechnology Research and Innovation Council, National Centre for Cell Science, NCCS Complex, Savitribai Phule Pune University Campus, Pune, India; ^2^Regional Centre for Biotechnology, Faridabad, India

**Keywords:** *Caenorhabditis elegans (C. elegans)*, GNAO1 encephalopathy, Gα_o_, G-proteins, mutations, disorders, neurotransmitter, signaling pathway

## Abstract

GNAO1 encephalopathies are a group of neglected genetic disorders primarily occurring due to *de novo* mutations in the Gα_o_ protein-encoding gene. This gene is reported to be highly conserved among *Caenorhabditis elegans (C. elegans)* and humans, with a sequence similarity of nearly 80%. The *C. elegans* model system simplifies studying signaling pathways involved in several neurotransmitters, including GPCR pathways. Therefore, using this model system to delineate downstream effectors and clinical targets to Gα_o_ can be highly advantageous. Mutations that cause GNAO1 encephalopathy can be easily replicated in genetically modified and transgenic *C. elegans* and validated by rescuing phenotypic defects, primarily locomotion and egg-laying defects in worms. Although there are recent technical advancements in understanding the interacting proteins, there are unclear and uncertain hypotheses that explain the effect of Gα_o_ mutations in humans. In terms of the clinical aspect of this disorder, there are no available approved diagnostic procedures to detect GNAO1 encephalopathy in the early stages of life. The present diagnostic procedures reiterate symptoms and overlap with other neurological symptoms, resulting in neglected data of cases. Therefore, here we provide an overview of past research and a perspective of future work, with the primary objective of focusing on GNAO1 encephalopathy and using the *C. elegans* model system to study these pathogenic variants.

## 1 The Gα_o_ signaling pathway is associated with GNAO1 encephalopathy

**FIGURE 1:**
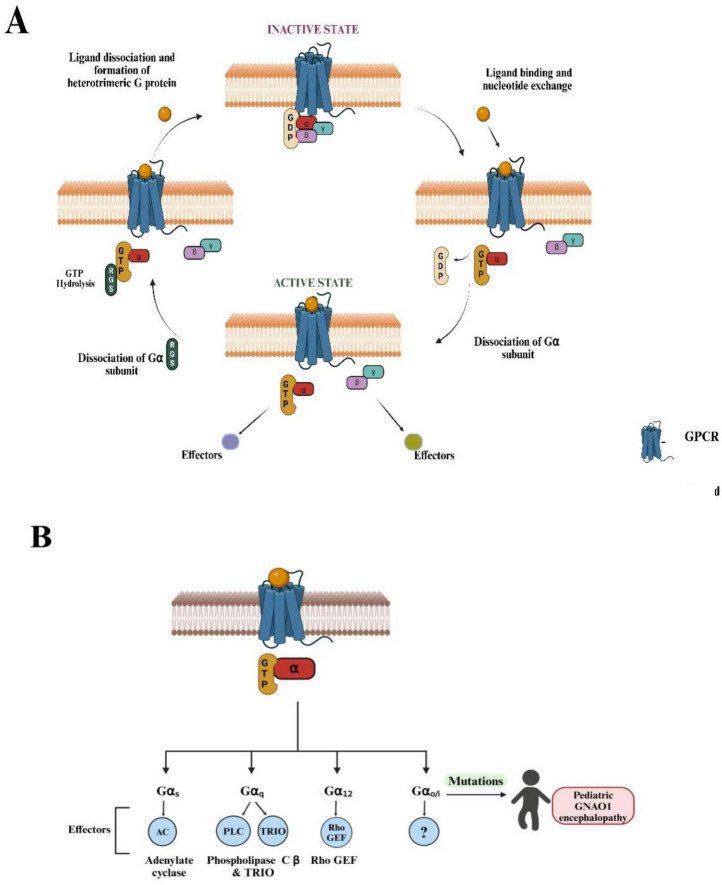
Overview of G-protein-coupled receptor (GPCR) signaling and known G-protein effectors. **(A)** The diagram illustrates the cyclic process of GPCR activation and G-protein signaling. (Top, left) In the inactive state, the GPCR is bound to an intracellular heterotrimeric G-protein, composed of Gα, Gβ, and Gγ subunits, with guanosine diphosphate (GDP) bound to Gα. (Top, right) Ligand binding to the extracellular domain of the GPCR induces a conformational change that facilitates GDP release and guanosine triphosphate (GTP) binding to Gα, activating the G-protein complex. (Bottom, right) The Gα subunit dissociates from the Gβγ dimer, and both subunits can potentially interact with downstream effectors, initiating intracellular signaling pathways. (Bottom, left) GTP hydrolysis by the regulator of G-protein signaling RGS proteins (regulators of G-protein signaling) leads to the reformation of the inactive heterotrimeric complex and terminates the signaling. This cycle repeats as long as the ligand remains bound to the GPCR. **(B)** Four distinct Gα subtypes (Gα_s_, Gα_q_, Gα_12_, Gα_o_) are shown, each modulating different downstream effectors: adenylate cyclase (AC) for Gα_s_, phospholipase-C β (PLCß) and TRIO (Trio Rho Guanine Nucleotide Exchange Factor) for Gα_q_, and Rho-GEF (Guanine Exchange Factor) for Gα_12_. The universal effector for Gα_o_ is currently unknown (denoted by “?”). Mutations in the *GNAO1* gene (OMIM: 615473, 617493), which encodes the Gα_o_ protein, are associated with pediatric GNAO1 encephalopathy, as highlighted on the right side of the diagram. These mutations can lead to severe neurological conditions, highlighting the clinical relevance of Gα_o_ in brain development and function. (Figures created with BioRender.com).

G-protein coupled receptors (GPCRs) are a major class of surface receptors, constituting about 5% of the genome. To date, humans have approximately 1,000 GPCRs classified, each with a unique and distinct function (Zhang et al., 2024). The major functions of GPCRs involve mediating most of the cellular responses to taste, olfaction,

and vision (Clapham and Neer, 1997). The prominent role of GPCRs is implicated in the fact that they bind to several ligands like neurotransmitters, ions, and hormones, and are majorly involved in the transmission of extracellular signals into intracellular responses. The implication of GPCRs lies in their ubiquity and diversity as they are involved in an inclusive range of biological functions from regulating blood pressure and heart rate to controlling immune responses and releasing hormones (Villaseca et al., 2022). GPCRs are coupled with G-proteins (guanosine-binding) proteins, which act as molecular switches, transmitting signals from activated cell surface receptors to intracellular proteins. In signal transduction, there are two primary types of G-proteins: monomeric GTP-binding proteins and heterotrimeric GDP-binding proteins (Xiao et al., 2023). The activation of G-proteins and their subsequent interaction with effector proteins mediate a wide range of cellular responses, including changes in enzyme activity, ion channel opening or closing, and modulation of intracellular signaling pathways (Wickman and Clapham, 1995). In its usual conformation, the G-proteins occur as heterotrimeric complexes involving the Gα, Gβ, and Gγ subunits. Neurotransmitter receptors stimulate Gα subunits to exchange bound GDP for GTP. Gα-GTP then separates from the Gβγ, and these activated G-protein subunits can evoke responses in the cell. Signaling is terminated when the Gα proteins hydrolyze their bound GTP, thus returning to the GDP-bound state and re-associating with Gβγ (Figure 1A; Jiang and Bajpayee, 2009). G-proteins are further classified into four main families: Gα_*s*_, Gα_*i/o*_, Gα_*q*_, and Gα_12_, each mediating different physiological responses depending on the tissue type and signal received (Hepler and Gilman, 1992). Among these, the Gα_*i/o*_ family, Gα_o_ (encoded by the *GNAO1* gene), is the most abundant G-protein found in brain tissue and controls both the development and adult physiology of the brain. It is particularly crucial for regulating neuronal signaling, specifically in neurodevelopment and synaptic transmission (Wettschureck and Offermanns, 2005). Recently, Gα_o_ has been characterized as an inducer of neuronal differentiation (Ju et al., 2014). In *C. elegans*, the homolog of the human Gα_o_ protein is GOA-1. This protein plays a vital role in inhibiting neurotransmitter release by negatively regulating synaptic vesicle exocytosis (Mendel et al., 1995). GOA-1 acts via several signaling pathways, including those involving diacylglycerol (DAG) and protein kinase C (PKC), modulating locomotion and egg-laying behaviors in *C. elegans* (Ségalat et al., 1995). Loss-of-function mutations in GOA-1 result in hyperactive neurotransmission that is reflected by hyperactive locomotion and increased egg-laying behavior due to dysregulated synaptic vesicle cycling (Miller et al., 1996). For the other Gα subunits, the downstream effector molecules are established using a combination of forward genetics and biochemical analysis (Buhl et al., 1995; Schade et al., 2005; Smrcka and Sternweis, 1993). However, Gα_o_ remains the only type of Gα protein in higher eukaryotes for which a universal effector molecule has not been identified (Figure 1B). Although Gα_o_ effector molecules have been identified in some model systems, these are not universal and have not been consistently reported in other systems (Purvanov et al., 2010; Savoia et al., 2018; Solis et al., 2017). For the other Gα subunits, the pathways and the effector molecules have been classified, but in the case of Gα_o_, it is unclear even after three decades of extensive research. This is a remarkable gap in our research knowledge since Gα_o_ constitutes 1% of the total membrane protein in the brain, making it orders of magnitude more abundant than other neural G-proteins (Muntean et al., 2021). Over the past two decades, many unsuccessful attempts have been made to classify the interacting effector molecules (signaling proteins) for Gα_o_ (Cuppen et al., 2003). The challenge in identifying Gα_o_ effectors through forward genetics in *C. elegans* could be attributed to several factors. First, the redundancy of multiple functionally similar effectors may mask the effects of mutations in single genes, making it difficult to observe changes in Gα_o_ signaling (Koelle, 2018). Second, if a Gα_o_ effector is vital for survival or reproduction, mutations could result in lethality or impair recovery of such mutants in genetic screens (Wang et al., 2022).

The α subunit of heterotrimeric G-proteins, Gα_o_, is encoded by the human *GNAO1* gene. These proteins couple with a variety of GPCRs, including dopamine, opioid, and serotonin receptors, among other crucial receptors, to perform significant neuromodulatory functions. Although various signaling events in which Gα_o_ is involved have been described, the systematic actions of Gα_o_ in the nervous system have not been fully understood. The term GNAO1 encephalopathy-related disorders, or GNAO1 encephalopathy, refers to a group of *de novo* GNAO1 mutations linked to neuro-developmental disorders, primarily developmental and epileptic encephalopathy 17 (also known as early infantile epileptic encephalopathy) (DEE) and neurodevelopmental disorder with involuntary movements (NEDIM) (Briere et al., 2023; Nakamura et al., 2013). GNAO1 encephalopathy has a wide range of developing phenotypes. Weakened movement, a hallmark of chorea, dystonia, and dyskinesia, is one of the core phenotypes, while others may include developmental delay and epilepsy (Feng et al., 2017). Some of the symptoms of GNAO1 encephalopathy, such as decreased mobility and seizure susceptibility, have been supported by the evaluation of GNAO1 disorder-associated mutations in mice. Examining pathogenic GNAO1 mutations in different model systems will help determine how these genetic disruptions affect mobility and potentially have conserved functional implications. Invertebrates, such as the nematode *C. elegans*, have a high degree of conservation of Gα_o_, and its ortholog, G-protein o-alpha subunit (GOA)-1, controls circuits for both locomotion and egg-laying (Collins et al., 2016). For evaluating the functional impact of pathogenic GNAO1 mutations, *C. elegans* is an appropriate *in vivo* system because of its reasonably well-defined GOA-1 genetics.

Efforts to describe pathogenic GNAO1 mutations at the molecular level have produced encouraging but contradictory findings in rodent and mammalian systems. The pathogenic mutations were classified as loss of function, gain of function, and normal function in an early work that used a heterologous cell-based assay to evaluate a pertussis toxin-insensitive variant of Gα_o_ (Wang et al., 2022). Most of the currently studied GNAO1 mutations cause loss of function, according to a recent assessment of rescued Gα_o_. Several mutations have been shown to behave as dominant negatives and counteract the transduction of GPCR signals (Briere et al., 2023; Di Rocco et al., 2023). *In vitro* results with G203R, R209C, and the less well-characterized G42R mutation are particularly noteworthy (Di Rocco et al., 2022; Di Rocco et al., 2023). These were later discovered to be loss of function and dominant negative after being first characterized as the gain of function or normal function. The causes and functional effects of GNAO1 pathogenic mutations are still unclear as a result of these conflicting findings. Investigating Gα_o_ function in the neurological system and creating therapeutic options for GNAO1 encephalopathy have attracted a lot of attention. Therefore, there is an excellent rationale for using *in vivo* models to study the behavioral effects of mutations associated with GNAO1 disorders. Here, we highlight the importance of studying GNAO1 mutations and effectors of Gα_o_ in the context of GNAO1 encephalopathy using *C. elegans* as the model system.

## 2 Current status of GNAO1 encephalopathy

### 2.1 GNAO1 encephalopathy: current treatments and clinical insights

GNAO1 encephalopathy remains considerably rare, with only a few sporadic cases reported globally, and to date, more than 350 cases have been reported (Sáez González et al., 2023; US Govt, 2024). However, the number can be an underrepresentation of cases, especially from developing countries, due to a lack of awareness and diagnostic challenges of genetic diseases. There is no available cure for GNAO1 encephalopathy-related disorders. A wide range of neurological issues have been linked to GNAO1-Related Disorders (RD), which include epilepsy, developmental delays, and hyperkinetic movement disorders (such as dystonia, chorea, and myoclonus) (Sáez González et al., 2023). These patients may have both chronic (baseline) and episodic (exacerbations) movement abnormalities, necessitating different treatment modalities. Pharmacological treatments for basal movement dysfunction, such as persistent dystonia and chorea, include gabapentin, benzodiazepines, clonidine, and tetrabenazine. These drugs aid in the treatment of chronic involuntary movements that interfere with normal daily activities. While benzodiazepines and clonidine deal with related symptoms involving anxiety and sympathetic dysregulation, tetrabenazine, in particular, is frequently used to reduce hyperkinetic movements (Danti et al., 2017; Domínguez Carral et al., 2024; Ling et al., 2022). Additionally, gabapentin enhances a patient’s quality of life by controlling pain and spasticity as an adjuvant (Akasaka et al., 2021). Conversely, a more rapid and forceful response is needed for treatment when patients have dyskinetic crises, which are exacerbations of their movements. Typically brought on by illnesses, emotional stress, or environmental factors, these episodes are marked by abrupt, severe manifestations of unusual movements. Drugs like benzodiazepines and clonidine are used to mitigate the severity of symptoms in these acute crises. The overall goal of managing GNAO1-RD is to reduce both acute exacerbations and baseline movement abnormalities with an assortment of pharmacologic medications. Additionally, avoiding consequences that can occur during dyskinetic crises is still crucial (Domínguez Carral et al., 2024). Improving both short-term and long-term results requires the creation of treatment plans customized to each patient’s needs. Despite of the comprehensive research, there are no FDA approved drugs for GNAO1. The ongoing and planned clinical trials are summarized in Table 1.

**TABLE 1:** Summary of clinical trials investigating GNAO1-associated disorders and therapeutic strategies.

NCT number	Title of the study	Status	Study type	Key objectives and summary	Relevance for repurposing/drug development
NCT04950946	GNAO1 natural history study	Unknown	Observational	Aims to collect natural history data to understand disease progression in GNAO1-related disorders.	Baseline data from this study could inform endpoints and stratification in future interventional trials, including drug repurposing efforts.
NCT06412653	Prospective pilot trial to address feasibility of DBS in pediatric GNAO1 patients	Recruiting	Interventional (single group)	Investigates the safety and feasibility of Deep Brain Stimulation (DBS) in children with GNAO1 mutations.	Can help establish DBS as a precision-targeted neurostimulation therapy. Results may guide combined therapeutic strategies (e.g., neuromodulation + pharmacotherapy).
NCT06912841	DBS match maker	Enrolling by invitation	Observational	Aims to match patients with appropriate DBS settings based on phenotypic data; supports personalized neuromodulation approaches.	Supports development of data-driven algorithms that may be used to optimize DBS and inform repurposing of neuroactive agents.
NCT06585605	Retrospective survey-based study in epilepsy-dyskinesia overlap syndromes	Recruiting	Observational	Collects survey-based data on epilepsy-dyskinesia overlap in genetic disorders such as GNAO1.	May reveal off-label use patterns and real-world effectiveness of antiepileptic or antidyskinetic drugs, highlighting repurposing candidates.
NCT06967727	Registry and natural history of epilepsy-dyskinesia syndromes	Not yet recruiting	Observational	Establishes a clinical registry to collect prospective data on disease trajectory and treatment response.	Critical for longitudinal tracking of drug responses and outcomes, which may uncover efficacy of FDA-approved drugs in new indications.

This table provides an overview of key ongoing or planned clinical trials related to GNAO1-associated neurodevelopmental and movement disorders. It includes both observational and interventional studies aimed at understanding disease progression, evaluating neuromodulation. These clinical investigations lay the groundwork for precision therapeutic strategies and future interventional trial design (ClinicalTrials.gov, 2025).

The other emerging area of treatment for genetic mutations is gene therapy, where the RNA interference (RNAi) approach has been widely explored. RNAi is the biological process that involves the silencing of gene expression mediated by the formation of double-stranded RNA in the system. This mechanism was discovered for the first time in the *C. elegans* model system (Fire et al., 1998). RNAi as a potential approach to gene therapy was described way back in 2003 (Caplen, 2003). For the first time, evidence supporting the successful RNAi-based gene therapy has been shown by targeted nanoparticles in human cancerous cell lines (Davis et al., 2010). These advancements have paved the way for RNAi-based therapeutic approaches for genetic diseases causing neuronal abnormalities like epilepsy and movement disorders. One such approach involves the strategy “Silence-and-replace” mechanism, where shRNA mediated by adeno-associated vector (AAV)-DJ serotype vectors in primary mouse neuronal cultures resulted in suppression of endogenous Gα_o_ (Lunev et al., 2022). Recent studies over the past five years have confirmed the dissection of GOA-1 function with *C. elegans* for *in vivo* RNAi as well as rescue approaches. Whole-organism RNAi feeding or injection effectively mimics GOA-1 loss-of-function (LOF) phenotypes. CRISPR-engineered worms bearing disease-associated hGNAO1 variants (e.g., S47G, A221D) show similar phenotypes, like hyperactive locomotion, premature egg-laying, and aldicarb hypersensitivity (Sáez González et al., 2023). Additionally, neuron-specific knockdown through the use of an NMD-based RNAi strategy and its targeted rescue in HSN neurons demonstrated that GOA-1 acts in a cell-autonomous way while regulating egg-laying behavior (Maher et al., 2013). Together, these strategies elucidate the study of LOF variants and enable the distinction of the hypomorphic alleles and GOF variants through the targeted RNAi rescue. This highlights the utility of *C. elegans* as a robust *in vivo* model for investigating the molecular and cellular mechanisms underlying GNAO1-related disorders. RNAi as a therapeutic approach to GNAO1 encephalopathies is an active area of research in GPCR biology. The potential of zinc salts as a treatment for GNAO1-associated disorders has been described in a few studies, following a progression from protein studies to human trials. The study includes previous findings on proteins, cells, and *Drosophila* models, with new data on mice and a human patient. Although zinc salts did not show toxicity to C215Y/+ mice, they did improve the behavioral defects. Also, a case report mentions that a human patient with a G203R mutation in Gα_o_ responded well to high-dose zinc therapy (Larasati et al., 2024).

### 2.2 GNAO1 encephalopathy: mutation analysis and phenotypic defects

**FIGURE 2:**
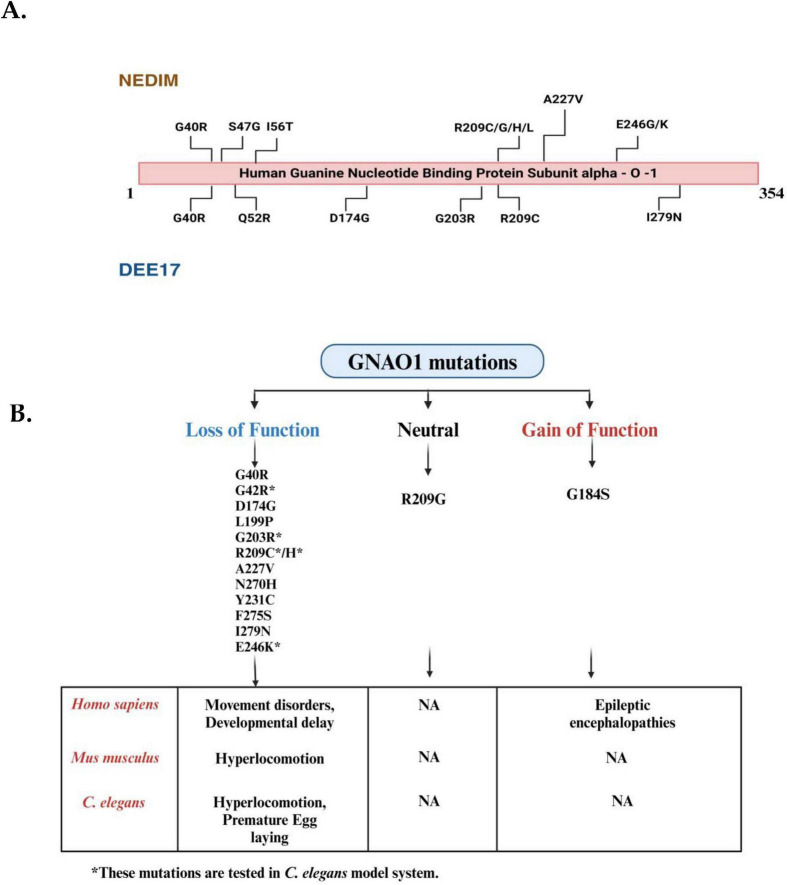
Mutational landscape of GNAO1 mutations associated with neurodevelopmental disorder with involuntary movements (NEDIM) and developmental and epileptic encephalopathy-17 (DEE17) syndromes and overview of their phenotypic effects in Homo sapiens, *Mus musculus*, and *Caenorhabditis elegans*. **(A)** The linear schematic represents the hGNA01 protein sequence (1-354 amino acids), highlighting the locations of disease-related mutations. Mutations identified in patients with NEDIM are shown above the protein, while mutations associated with DEE17 are indicated below the protein. Key mutations include G40R and A227V, among others, with multiple variations at residue R209. **(B)** Mutations in the *GNAO1* gene are categorized into three groups: loss-of-function mutations (left, blue), neutral mutations (center, black), and gain-of-function mutations (right, red). Each category lists relevant mutations based on their functional impact. Loss-of-function mutations include G40R, G42R, D174G, L199P, A227V, N270H, Y231C, F275S, G203R, R209C/H, E246K and I279N, while gain-of-function mutations include G184S.R209G represents neutral mutations (Mutations present only in the coding region have been depicted here). The bottom section highlights the phenotypic changes associated with these mutations across different species, including *Homo sapiens* (humans), *Mus musculus* (mice), and *C. elegans* (nematodes). Loss-of-function mutations are associated with movement disorders and developmental delays in humans, whereas gain-of-function mutations cause epileptic encephalopathies. In *Mus musculus*, loss-of-function mutations result in hyperlocomotion, but no phenotypic data are available for gain-of-function mutations. In *C. elegans*, loss-of-function mutations lead to hyperlocomotion and premature egg-laying, while no conclusive data is available for the gain-of-function mutations (The mutations listed are for representative purposes only; they do not mean to be already screened in the *C. elegans* model system). (Figures created with BioRender.com).

Recent studies suggest that silencing the pathogenic variant at the genetic level can prevent phenotypic defects (Klementieva et al., 2024). All the major mutations occurring in *GNAO1* have been represented in Figure 2A, which fall under two categories of neurodevelopmental disorders, i.e., NEDIM and DEE17. Among these, studies indicate the occurrence of more frequent G203R, R209C, or E246K mutations in GNAO1 encephalopathies (Di Rocco et al., 2022; Di Rocco et al., 2023; Silachev et al., 2022). It has been shown in mouse models that contain the G203R pathogenic mutant of GNAO1 that is neonatally lethal even in heterozygous conditions, but the same heterozygous variant has been successfully silenced using RNA interference in cell-based assays (Solis et al., 2024). The mutations in the GTP binding region of Gα_o_ are further classified as loss of function (loss of GDP exchange activity) and gain of function (hyperactive GTP-binding capacity), affecting the levels of activated Gα_o_. As discussed in Figure 2B, the defects in humans and mice are indistinguishable, making it difficult to assess the outcomes of the mutations. In contrast, *C. elegans* model system, the phenotypic defects are evident with distinguishable locomotory and egg-laying defects. These findings collectively suggest the need for a simpler yet conserved model system to find the potential treatment for GNAO1 encephalopathy. *C. elegans* is an imperative *in vivo* system for assessing the functional genetic effects of GNAO1 pathological mutations. Prior research has shown evidence that *C. elegans* is useful in investigating the molecular genetic basis of neurodevelopmental disorders (Table 2; Dexter et al., 2012; Lee et al., 2016; Locke et al., 2006). Indeed, given the growing number of GNAO1 pathogenic variations discovered to date, *C. elegans* would be the ideal model organism for functional assessment (Saitsu et al., 2016).

**TABLE 2:** Summary of GNAO1 coding and non-coding variants studied in human patients and their orthologous phenotypes in *Caenorhabditis elegans.*

No.	Mutation in the Gα_o_ Gene	Amino-acid change	Description of mutation	Phenotype in humans	Phenotype in *C. elegans*	References
1	c.625C > T	p.(Arg209Cys)	Missense variant	Developmental delay, intellectual disability, and severe chorea, associated with the later onset of complex partial seizures	Hyperactive locomotion and egg-laying defects	Danti et al., 2017; Di Rocco et al., 2022; Saitsu et al., 2016
2	c.139A > G	p.(Ser47Gly)	Missense variant	Novel; Severe ID/DD, hypotonia, movement disorders, seizures	Hyperactivity and enhanced egg laying	Danti et al., 2017; Di Rocco et al., 2022
3	c.723 + 1G > A	Not defined	Intronic variant	Novel; Severe ID/DD, hypotonia, movement disorders, seizures	Nd	Danti et al., 2017
4	c.167T > C	p.(Ile56Thr)	Missense variant	Novel; Mild ID focal epilepsy, movement disorder	Nd	Danti et al., 2017
5	c.118G > C	p.(Gly40Arg)	Missense mutation	Novel	Nd	Danti et al., 2017
6	c.118G > A	p.(Gly40Arg)	Missense mutation	Infantile-onset epilepsy	Nd	Law et al., 2015
7	c.737A > G	p.(Glu246Gly)	Missense variant	Novel; dystonia	Nd	Danti et al., 2017
8	c.736G > A	p.(Glu246Lys)	Not defined	Developmental delay, intellectual disability, and a paroxysmal movement disorder	Hyperactive locomotion and egg-laying defects	Danti et al., 2017; Di Rocco et al., 2022
9	c.836T > A	p.(Ile279Asn)	*De novo* missense mutation	Ohtahara syndrome	Nd	Nakamura et al., 2013
10	c.521A > G	p.(Asp174Gly)	Missense mutation, somatic mosaic	Ohtahara syndrome	Nd	Nakamura et al., 2013
11	c.572_592 del	p.(Thr191_Phe197del)	Deletion	Ohtahara syndrome, dystonia	Nd	Nakamura et al., 2013
12	c.607G > A	p.(Gly203Arg)	*De novo* missense mutation	Epileptic encephalopathy, severe chorea, athetosis	Nd	Nakamura et al., 2013
13	Not defined	p.Ala338del	*De novo*	Novel; hypotonia	Nd	Kim et al., 2020
14	Not defined	p.Ala227Val	*De novo*	hypotonia	Nd	Kim et al., 2020; Saitsu et al., 2016
15	Not defined	p.Ala301del	*De novo*	Hypotonia	Nd	Kim et al., 2020
16	Not defined	p.Arg209His	Maternal mosaicism	Chorea	Nd	Danti et al., 2017; Kim et al., 2020; Kulkarni et al., 2016
17	Not defined	p.Arg206Leu	*De novo*	Developmental delay	Nd	Kim et al., 2020
18	c.111-113del	p.Leu39del	Not defined	Motor and cognitive delay, Severe intellectual deficiency, EIDEE, Cortical atrophy	Nd	Kim et al., 2020
19	c.118G > C	p.G40R	Not defined	Seizures, hypotonia	Nd	Danti et al., 2017; Law et al., 2015
20	c.709G > A	p.Glu237Lys	*De novo*	Hyperkinesia, cerebral palsy, Movement disorder	Nd	Graziola et al., 2021; Koy et al., 2018; Ling et al., 2022; Okumura et al., 2018; Waak et al., 2018
21	c.626 G > A	p.R209H	Not defined	Dystonia, ataxia	Nd	Dhamija et al., 2016; Menke et al., 2016
22	c.692A > G	p.Y231C	Not defined	Myoclonic seizure, Hypotonia	Nd	Talvik et al., 2015
23	c.836T > A	p.I279N	Not defined	Chorea, akathisia, hypotonia	Nd	Nakamura et al., 2013
24	c.118G > T	p.G40W	Novel	Focal seizures, DEE	Nd	Kelly et al., 2019
25	c.119G > A	p.G40E	Novel	Myoclonic, tonic seizures, GTC, spasms	Nd	Kelly et al., 2019
26	c.620C > A	p.S207Y	Novel	Developmental delay, dystonia	Nd	Kelly et al., 2019
27	c.662C > A	p.A221D	Novel	Developmental delay, dystonia	Hyperactive locomotion and increased egg laying	Di Rocco et al., 2023; Kelly et al., 2019
28	c.818A > T	D273V	Novel	Seizures, dyskinesia, hypotonia	Nd	Kelly et al., 2019
29	c.871T > A	p.Y291N	Novel	Focal seizures	Nd	Kelly et al., 2019
30	c.1030_1032delATT	p.I344del	Novel	Movement disorder, dystonia, chorea	Nd	Kelly et al., 2019
31	c.1046_1055del10ins10	p.R349_G352delins QGCA	Novel	Facial dyskinesia, tremor, developmental delay	Nd	Kelly et al., 2019

Each entry gives the nucleotide change (c.-notation) and predicted amino-acid alteration or indel, along with variant classification (missense, intronic, deletion, somatic mosaicism) and the range of clinical presentations seen in affected individuals: ranging from early-onset epileptic encephalopathies and developmental delay to hyperkinetic movement disorders (chorea, dystonia) and hypotonia. Where there have been functional assays in *C. elegans*, equivalent nematode phenotypes (e.g., hyperactive locomotion, increased egg-laying) are shown; variants without *in vivo* modeling are marked as “not assessed.” New or *de novo* alleles are emphasized, and primary literature sources are referenced for each variant to enable a full genotype–phenotype correlation. Nd, not determined.

## 3 *C. elegans* as a potential model system to study *GNAO1* mutations

GNAO1 mutations remain highly understudied because of the unavailability of human subjects due to the extremely low number of reported cases and technical difficulties, permissions, and ethical concerns associated with obtaining human samples. To mitigate the mutations responsible for GNAO1 encephalopathy, researchers have traditionally been using rodent models, particularly mice. There were reported studies of unsuccessful attempts in mouse models to study GNAO1-related disorders. Further, in the quest to find a simpler model system, an attempt was made to replace *Drosophila melanogaster’s* Gα_o_ with human *GNAO1* successfully. While approximately 75% of human genes associated with diseases have counterparts in *Drosophila*, the proteins they produce differ in exon sequences by alternative splicing, which may limit the translation of findings from the *Drosophila* model to human conditions, particularly in drug discovery (Savitsky et al., 2020). These reports establish the need for the use of the alternative model system with the ease of genetic manipulation and evident phenotypic defects. *C. elegans* has been established as a valuable experimental model for understanding GNAO1-related disorders and exploring potential treatments. The researchers created two genetically modified strains with mutations at key positions Glu246 and Arg209, known to be crucial in Gα_o_ (Di Rocco et al., 2022; Di Rocco et al., 2023). These loss-of-function (LOF) mutations caused varied reductions in Gα_o_-mediated signaling, resulting in excessive neurotransmitter release from different neuron types (Table 2). This led to hyperactive behaviors like increased egg-laying and movement. Key scoring metrics for hyperactive egg laying included the total number of unlaid eggs, frequency of egg-laying bursts (e.g., bursts every ∼20 s during active states), and inter-burst intervals (Ravi et al., 2021). Notably, single-copy mutations demonstrated cell-specific dominant-negative effects in neurons, determined by the specific altered residue. Similar to earlier mutants S47G and A221D, caffeine effectively mitigated the excessive movement in animals with the R209H and E246K mutations, implying caffeine’s independent action from the mutation type. In contrast, the adenosine receptor antagonist “Istradefylline” was effective in R209H animals but not in E246K worms, suggesting caffeine’s multifaceted mode of action (Di Rocco et al., 2023). Overall, these findings deepen our understanding of the disease mechanisms and provide more evidence for caffeine’s potential efficacy in managing dyskinesia linked to GNAO1 loss-of-function mutations. In contrast, models involving the gain-of-function (GOF) mutation of Gα_o_, (G203R) or Gα_o_(209C) in striatal neurons led to impaired locomotor behavior in mice, which provides insights into G-protein functioning and its mutants (Baik et al., 1995), but its relevance as a disease model is still not clear. Interestingly, hyperactivity seen in C215Y/+ mice is associated with the motor cortex rather than anatomical defects in the striatum. These findings highlight the complexity of modeling GNAO1 encephalopathy and the importance of accurately replicating the disease phenotype in animal models for meaningful research and therapeutic development. A similar result of abnormal locomotor behavior was seen in rodents in previous studies when mutations linked to disorders were tested by CRISPR editing of the native GOA-1/Gα_o_ locus in *C. elegans*, suggesting that there is a strong association of how pathogenic variations of GNAO1 impact Gα_o_ function in living organisms (Polikarpova et al., 2023). This association is reasonable as the molecular mechanisms underlying locomotor movements in mouse models and *C. elegan*s are comparable. The *C. elegans* motor circuit, which consists of excitatory cholinergic and inhibitory GABAergic motor neurons, as well as GABAergic striatal neurons (dMSNs and iMSNs), which are responsible for motor coordination in mice, is responsive to dopaminergic modulation (Chase et al., 2004). In both cases, loss of Gα_o_ function results in hyperactive locomotion. Furthermore, Gα_o_ signaling serves to impair neuronal activity in both *C. elegans* and mammals (Jiang and Bajpayee, 2009). Determining the genetic processes by which GNAO1 variations impact locomotor activity is made possible by the power of the *C. elegans* model system, which may also hold the key to resolving unresolved mechanistic questions about the molecular basis of GNAO1 encephalopathy. Additionally, *C. elegans* may be used for genetic and small chemical screens that target GNAO1 and may potentially be developed as an *in vivo* platform that can assess a large number of GNAO1 variations. These conserved features have proven advantageous in the study of GNAO1 variants in *C. elegans*.

The spectrum of GNAO1 encephalopathy is wide and continually evolving. As mentioned previously, the central aspect of this condition is compromised motor function, along with epilepsy and developmental delay, which are prevalent features of this phenotype (Sáez González et al., 2023). Gα_o_, being a major G-protein, is expressed across many brain regions, including the cerebellum, wherein it is cellularly localized within the Purkinje fibers, basket, and stellate cells, which inhibit GABAergic interneurons and Golgi neuronal cells. It forms connections with various crucial G-protein coupled receptors (GPCRs) such as GABA-β, α2 adrenergic, adenosine A1 (A1R), and dopamine D2 (D2R) receptors. These receptors are essential for controlling neurotransmitter release, movement, and neural development functions (Muntean et al., 2021). There are numerous postulated downstream targets in the signaling pathway of Gα_o_, as well as the other members of the Gα family. Many of the targets affected by Gα_o_ signaling are also implicated in disorders related to movement. Mutations in other signaling molecules remain understudied but are of great importance. For example, genetic mutations in ADCY5, the gene responsible for encoding adenylyl cyclase type 5, have been identified in patients with dyskinesia and dystonia (Mencacci et al., 2015). The prevailing characteristics among patients harboring GNAO1 mutations are hypotonia and developmental delay, irrespective of their clinical presentation or biochemical traits. After these, chorea and dystonia are the subsequent most frequent observations (Kelly et al., 2019). While a notable proportion of individuals exhibit atypical EEG or MRI results, fewer than fifty percent of those with GNAO1 mutations displayed distinctly aberrant EEG patterns, primarily among patients with epilepsy and loss-of-function (LOF) mutations. This diversity, encompassing variations in clinical presentation and impact on brain structure/function, suggests an involvement of both neurodevelopmental changes and disruptions in functional signaling. The latter factor seems to be more prominent in patients with gain-of-function (GOF) mutations, who exhibit fewer indications of structural brain abnormalities and display partial positive responses to drug interventions. The potentially uncertain underlying factors contributing to GNAO1-related movement disorders can be elucidated by examining GNAO1 signaling. One potential pathway involves the canonical regulation of cAMP by Gα_o_, which can be facilitated by Gα_o_ or the liberated Gβγ (Muntean et al., 2021). Notably, mutations in ADCY5, responsible for encoding an AC protein that generates cAMP, also lead to movement abnormalities in human patients. The disruption of cAMP signaling has been linked to impaired brain function (Mencacci et al., 2015). Consequently, disturbances in cAMP levels could perturb the delicately balanced neurodevelopmental system, suggesting that the operation of Gα_o_ via the cAMP pathway might be important in movement disorders, yet the downstream effectors have not been studied.

A second theoretical foundation for GNAO1-associated movement disorders pertains to Gα_o_’s involvement in governing neurotransmitter release. The deficiency of crucial neurotransmitters like catecholamines (such as dopamine, epinephrine, and norepinephrine) and serotonin has been extensively studied in the context of movement disorders or seizures. Gα_o_’s presynaptic role in regulating neurotransmitter release presents another potential avenue in the understanding of movement disorder etiology (Ravi et al., 2021).

A third conceivable perspective, centered on developmental considerations, involves potential alterations in the maturation of neurons, a process vital during appropriate stages of neurological development. Consequently, children with developmental abnormalities might display irregular behaviors. Notably, most individuals with GNAO1-linked movement disorders also suffer from significant developmental delays. Morphologically, MRI scans may reveal widespread atrophy and delayed myelination. In general, genetic factors account for around 40% of cases involving developmental delay, including intellectual disability (Mercimek-Mahmutoglu et al., 2015). Control over cAMP levels and neurotransmitter release can influence ongoing neural functions as well as neurological development. Within this framework, GNAO1-associated movement disorders could arise from disruptions in either or both processes. The former scenario would likely be more open to therapeutic interventions compared to the latter.

**FIGURE 3:**
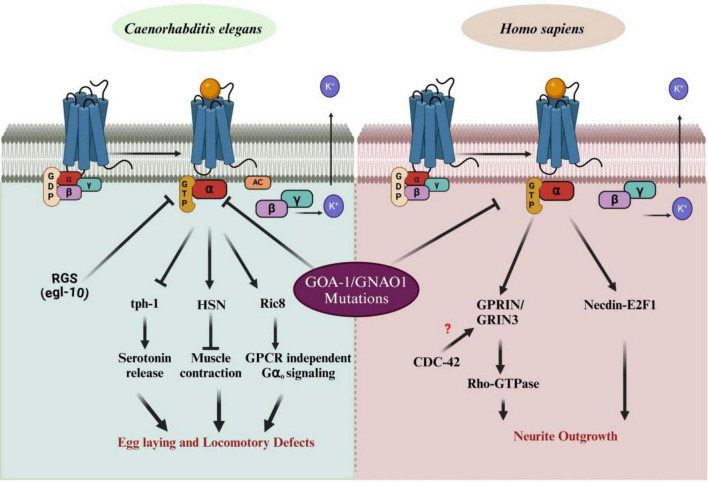
Impact of GNAO1 mutations on G-protein signaling pathways in *C aenorhabditis elegans* and Homo sapiens. The **left panel** illustrates normal G-protein signaling pathways in *C. elegans*, showing the interaction of Gα [bound to guanosine diphosphate (GDP)] with Gβγ upon G-protein-coupled receptor (GPCR) activation. GNAO1 (Gα) regulates processes such as serotonin release, muscle contraction, and egg-laying behavior via downstream effectors such as tph-1 and Ric-8. Mutations in GNAO1 lead to defects in egg laying and locomotion. The **right panel** demonstrates the G-protein signaling in *Homo sapiens.* GNAO1 mutations disrupt neurite outgrowth by affecting the interaction with Rho-GTPase signaling, Necdin-EF1, and the GPRIN/GRIN3 complex, with potential involvement of CDC-42, which remains unclear. Arrows indicate pathways affected by GNAO1 mutations, contributing to abnormal neuronal growth and function. RGS, regulator of G-protein signaling; tph-1, tryptophan hydroxylase; Ric8, resistance to inhibitors of cholinesterase; GRIN-1, glutamate inotropic receptor NMDA subunit -1; CDC-42, cell division control protein – 42; AC, adenylyl cyclases (Figures created with BioRender.com).

The conserved nature of G-protein signaling across species enables us to use the *C. elegans* model system, while also highlighting the specific downstream effectors corresponding to phenotypic defects. GNAO1 mutations significantly impair normal biological functions, manifesting as distinct phenotypes: locomotory defects in *C. elegans* and defective neuronal development in humans. Understanding these pathways can provide critical insights into the molecular mechanisms underlying GNAO1-related diseases and may offer therapeutic targets for treating associated disorders. In *C. elegans*, the Gα_o_ protein is involved in several signaling cascades. Upon activation, Gα dissociates from the Gβγ subunits, initiating a downstream signaling cascade that regulates key biological processes. These include serotonin release via tph-1, muscle contraction through the hermaphrodite-specific motor neuron (HSN), and GPCR-independent signaling through Ric8 (Campagna et al., 2023; Miller and Rand, 2000), which mediates Gα signaling even in the absence of receptor activity. The regulator of G-protein signaling (RGS) protein, egl-10, modulates Gα activity by accelerating GTP hydrolysis (Koelle, 1997), returning Gα to its inactive state bound to GDP. Mutations in *GNAO1* disrupt these pathways, resulting in egg-laying and locomotory defects in *C. elegans*, highlighting the critical role of G-protein signaling in neural and muscular functions. In *Homo sapiens*, mutations in GNAO1 are linked to neurological disorders, including epilepsy and movement disorders. Similar to *C. elegans*, Gα dissociates from Gβγ upon activation by GPCRs. However, in humans, GNAO1 mutations impact neuronal signaling pathways involved in neurite outgrowth. Specifically, these mutations influence Rho-GTPase signaling, which is modulated by GPRIN (G-protein-regulated inducer of neurite outgrowth) (Savoia et al., 2018). These proteins possibly interact with GNAO1 and Rho-GTPase via the CDC-42 pathway (Taira et al., 2024), which regulates cytoskeletal dynamics essential for neurite outgrowth and neuronal morphology. Additionally, the interaction between GNAO1 and Necdin-E2F1 modulates cell cycle regulation, further affecting neuronal differentiation (Ju et al., 2014). Defects in these pathways due to GNAO1 mutations result in impaired neurite development, contributing to the observed neurological phenotypes in patients (Figure 3). A clear picture of all the events is still unknown, opening a plethora of opportunities in the field of GNAO1 biology.

### Conclusion and perspective

The study of GNAO1 encephalopathy, a genetic disorder caused by mutations in the *GNAO1* gene, encoding the Gα_o_ subunit of G-proteins, presents significant challenges due to the complexity of G-protein-coupled receptor (GPCR) signaling pathways in the nervous system (Solis et al., 2017). Gα_o_, a highly abundant membrane protein in the brain, plays a critical role in neuro-modulation by interacting with various GPCRs, such as dopamine, serotonin, and opioid receptors (Muntean et al., 2021). This genetic disorder manifests in patients with a wide-ranging spectrum of symptoms, including developmental delays, epilepsy, involuntary movements, and other motor dysfunctions (Briere et al., 2023). The exact mechanisms underlying these phenotypes remain poorly understood. While mammalian models have provided some insight into the effects of GNAO1 mutations, they often present conflicting results, making it difficult to derive conclusive findings. In this context, *C. elegans*, a nematode with a well-conserved G-protein signaling pathway similar to that in humans, has emerged as an ideal *in vivo* model for studying the functional impacts of *GNAO1* mutations. *C. elegans* Gα_o_ (GOA-1) protein is highly homologous to human GNAO1, making it a valuable tool for assessing the consequences of mutations on locomotion, neurotransmitter release, and other nervous system functions. Several advantages contribute to *C. elegans* being the preferred model system for GNAO1 research. The simplicity of its neural circuit, coupled with its highly conserved genetic pathways, allows researchers to effectively replicate the mutations observed in human GNAO1 encephalopathy. They can study their effects on worms’ behavior, such as locomotion and egg-laying, which mirror the impaired movement and motor dysfunctions seen in human patients. Previous studies in rodent models have confirmed the involvement of Gα_o_ in movement disorders, but *C. elegans* offers the added benefit of being able to dissect the molecular mechanisms of these mutations with greater precision and at a faster pace (Silachev et al., 2022). For example, recent research using *C. elegans* has demonstrated that mutations in key residues, such as Glu246 and Arg209, lead to hyperactive behaviors like excessive movement and egg-laying, reflecting the overactive neurotransmitter release seen in patients with GNAO1 mutations (Di Rocco et al., 2023). The ability to observe these phenotypic changes in real-time makes *C. elegans* an invaluable model for understanding how specific mutations disrupt Gα_o_-mediated signaling pathways. In terms of therapeutic research, *C. elegans* has proven instrumental in identifying potential treatments for GNAO1 encephalopathy. For instance, studies have shown that caffeine can alleviate the hyperactive phenotypes caused by certain GNAO1 mutations, offering a potential avenue for pharmacological intervention (Di Rocco et al., 2022). This finding highlights the multifaceted nature of caffeine’s action, as it was effective across different mutations, suggesting that it may act on multiple pathways involved in Gα_o_ signaling (Di Rocco et al., 2022; Lee et al., 2016). Similarly, drugs like oxcarbazepine, traditionally used to treat epilepsy, have shown promise in managing movement disorders associated with GNAO1 mutations, further emphasizing the utility of *C. elegans* in pre-clinical drug screening (Ling et al., 2022). These findings highlight the potential of *C. elegans* not only as a model for understanding the genetic basis of GNAO1 encephalopathy but also as a platform for discovering novel therapeutic approaches, leading to several clinical trials (Table 1). Beyond its use in studying movement disorders, *C. elegans* offers a unique opportunity to investigate the role of GNAO1 mutations in epilepsy, a common feature of GNAO1 encephalopathy. Although much of the research has focused on the motor dysfunctions caused by GNAO1 mutations, seizures are significant and often life-threatening symptoms of this disorder. *C. elegans’* well-characterized nervous system and responsiveness to various neurotransmitters, including GABA and dopamine, make it an ideal system for studying the mechanisms of seizure induction and progression. This model has already been used to study seizure-like activity in other genetic disorders, and its application to GNAO1 research could provide new insights into how these mutations increase seizure susceptibility (Table 2). RNAi-based therapies for GNAO1 variants show promise in *C. elegans* due to its robust systemic RNAi machinery, but their translation to mammalian systems faces challenges, including incomplete gene silencing, potential off-target effects raising safety concerns, and inefficient delivery methods like viral vectors (e.g., AAV-DJ), which are invasive and suboptimal for deep brain regions or early developmental stages (Di Rocco et al., 2023). Similarly, caffeine effectively suppresses hyperactive phenotypes across multiple GNAO1 variants (e.g., S47G, A221D, R209H) in *C. elegans*, likely via adenosine receptor antagonism, but its precise molecular mechanism remains unclear, with variant-specific efficacy noted for selective A2A antagonists like Istradefylline (Di Rocco et al., 2022). The lack of dose-response studies, EC50 data, or pharmacokinetic modeling in *C. elegans*, coupled with poorly characterized caffeine metabolism, limits its therapeutic potential. Future research should focus on receptor-binding assays to confirm adenosine receptor involvement, dose-response profiling in *C. elegans* and mammalian models, exploration of adenosine analogs for selectivity, and neuron-specific studies using cell-type–specific promoters and calcium imaging to enhance translatability and therapeutic precision.

While *C. elegans* provides a powerful model for studying GNAO1 encephalopathy, there remain significant challenges in fully elucidating the downstream pathways through which Gα_o_ mutations exert their effects. The pathways involving GNAO1 are intricate, with numerous interactions between GPCRs, G-proteins, and their downstream effectors, many of which remain undefined. Moreover, the clinical diagnosis of GNAO1 encephalopathy is often delayed due to the overlap of symptoms with other neurological disorders, further complicating efforts to understand the full spectrum of this disease. Future research in *C. elegans* will need to focus on mapping these pathways and identifying specific molecular targets that could be used for early diagnosis and intervention. Extensive drug screening can be performed using *C. elegans* model system using a three-tier strategy. The primary screen will employ high-throughput behavioral assays such as egg-laying, locomotion, and feeding to identify compounds that produce physiological effects in both wild-type and mutant strains. Compounds identified in this phase will move to secondary validation, which will include genetic interaction studies such as epistasis analysis with known signaling mutants (e.g., EGL-30, GSA-1), along with phenotypic confirmation across developmental stages to assess consistency and specificity. Finally, the tertiary characterization phase will involve molecular and cellular assays to uncover mechanisms of action and confirm target engagement. This will include reporter gene analysis, neuronal imaging, and transcriptomic profiling. Although the significance of comprehensive omics-based approaches, such as global transcriptomics under stress or drug exposure, is well recognized. These approaches are beyond the current scope and aims of this study. Consequently, they have not been included or discussed in the manuscript.

In conclusion, *C. elegans* serves as an invaluable model for studying the molecular and phenotypic consequences of GNAO1 mutations. Its conservation of Gα_o_ signaling pathways, combined with its genetic tractability, provides a unique platform for both basic research and therapeutic development. By utilizing *C. elegans*, researchers can gain deeper insights into the mechanisms underlying GNAO1 encephalopathy and develop targeted treatments that address the complex interplay of motor dysfunctions, seizures, and developmental delays characteristic of this disorder. As research continues to advance, *C. elegans* will undoubtedly play a crucial role in unraveling the mysteries of GNAO1-related neurological disorders and improving outcomes for affected individuals.
